# Antinociceptive effect of methanolic extract and alkaloid fractions of *Berberis integerrima* root in animal models

**Published:** 2018

**Authors:** Valiollah Hajhashemi, Foroogh Fahmideh, Mostafa Ghanadian

**Affiliations:** 1 *Department of Pharmacology and Isfahan Pharmaceutical Sciences Research Center, School of Pharmacy and Pharmaceutical Sciences, Isfahan University of Medical Sciences, Isfahan, Iran*; 2 *Department of Pharmacognosy, School of Pharmacy and Pharmaceutical Sciences, Isfahan University of Medical Sciences, Isfahan, Iran *

**Keywords:** Berberis integerrima, Writhing test, Formalin test, Hot plate test, Antinociceptive

## Abstract

**Objective:**

*Berberis vulgaris* has antioxidant, hepato--renal protective, antibacterial, lipid lowering, anti-inflammatory and antinociceptive activities. The genus *Berberis*, has another member called *Berberis* *integerrima* which has not been studied for antinociceptive activity and therefore, this study was aimed to examine the antinociceptive effect of total extract and alkaloid fractions of *Berberis integerrima* root in mice.

**Materials and Methods:**

Methanolic total extract and alkaloid fractions of the plant namely, fractions A, B, C and D were prepared according to standard methods. Male Swiss mice (20-25 g) were used (n=6 in each group). Acetic acid-induced writhing, formalin and hot-plate tests were used to assess the antinociceptive activity. In hot plate and formalin tests, morphine (10 mg/kg, i.p.) and in acetic acid test, indomethacin (10 mg/kg, i.p.) were used as reference drugs.

**Results:**

The total extract and fractions A, B and D significantly reduced abdominal twitches in acetic acid test and licking behavior of both acute and chronic phases of formalin test. In hot-plate test, morphine as the standard drug demonstrated significant antinociception while the plant extract and fractions were ineffective. The dose of 5 mg/kg of fraction C showed slight analgesia only in acetic acid test and a dose of 10 mg/kg caused severe toxicity and even death in some animals.

**Conclusion:**

*Berberis integerrima* total extract and its alkaloid fractions showed antinociceptive effect and it seems that the mechanism of this action is peripherally mediated since they were effective in formalin test and acetic acid- induced writhing but not in hot-plate test.

## Introduction

Today, the tendency of people to use medicinal herbs is increasing (Ekor, 2013 ▶). Berberis commonly known as barberry is a large genus belonging to *Berberidaceae* family and is a well-known medicinal plant in Iran (Mokhber-Dezfuli et al., 2014 ▶). The most well-known Berberis species are *Berberis crataegina, Berberis integerriam, Berberis khorasanica, Berberis orthobotrys, Berberis vulgaris, and Berberis thunbergii *(Fatehi et al., 2005 ▶). 

*Berberis vulgaris* and *Berberis integerrima* (synonym to *Berberis densiflora*), called "Zereshk" and "Zereshk Kuhi" in Persian language, respectively grow in Iran. *B. vulgaris* grows abundantly in the mountainous regions of north-east of Iran (Khorasan province) while *B. integerrima* is distributed in the south (Alemardan et al., 2013 ▶; Zarei et al., 2015 ▶). Different parts of Berberis such as leaf, fruit, bark and root have shown various pharmacological properties (Imanshahidi and Hosseinzadeh, 2008 ▶). 

Worldwide,* Berberis* species are used as a folk remedy for treatment of some diseases like jaundice, rheumatism, lumbago and cardiovascular diseases and also to reduce fever, vomiting during pregnancy, and kidney and gall balder stones (Fatehi et al., 2005 ▶; Srivastava et al., 2015 ▶). Studies have shown that the main chemical compounds in the root and bark of Berberis are alkaloids and the most important alkaloid is berberine that has many therapeutic properties such as antioxidant, hepato-renal protective, antibacterial, reduce lipid, anti-inflammatory (used for treatment of some diseases like rheumatism and to reduce fever) and analgesic activities. 

*B. integerrima* is widely used by traditional medicine for treatment of jaundice, blood pressure, diabetes, and fever (Amiri et al 2014 ▶). While most researches have focused on *B. vulgaris*, only few studies have investigated biological and pharmacological activities of *B. integerrima* (Alimirzaee et al., 2013 ▶; Jamshidzade et al., 2006 ▶).

Since some species of *Berberis *like *crataegina,* *aristata* and *vulgaris* have shown anti-inflammatory, analgesic and antipyretic properties (Akhter et al., 1977 ▶; Ivanovska et al., 1996 ▶; Yesilada and Kupeli, 2002 ▶), this study was aimed to evaluate the analgesic effect of the extract and various alkaloid fractions of *B.* *integerrima* in mice.

## Materials and Methods

**Plant material and Extraction**

*B. integerrima *roots were collected in autumn 2016, form Zagros mountains, in Shamsabad valley (besides Borujen-Lordegan road) about 60 km south, Chaharmahal va Bakhtiari province, Iran at 2300 meters above sea level. It was identified by Prof. Hojjatollah Saeidi, from Department of Biology, Faculty of Science, University of Isfahan, Iran, where a voucher specimen was deposited.

Air-dried roots (2000 g) were powdered using an electrical mill (mesh number 100), and extracted by a classical percolation method using methanol. Plant material was soaked in solvent for 3 days in percolation vessel, and then allowed to percolate at a flow of about 3 ml/min, at room temperature for 4 days (a total of 10 liter solvent was used). 

Methanol extract was filtered and evaporated by rotary evaporator under reduced pressure at 40 ºC (85 g). Alkaloidal constituents were extracted as described by Rafeal Suau (Suau et al., 1998 ▶). As a general rule, alkaloids in their free state are soluble in organic solvents and only slightly soluble in water. They are soluble in water when ionized in acidic solutions. Their basicity may also differ greatly depending on the number and availability of the lone pair of electrons on the nitrogen atom in heterocycle rings. Therefore, for extraction of alkaloids, the extract syrup was first treated by diluted HCL (5 %, 2 L) and filtered. The clear filtrate solution was then allowed to precipitate at 4 ºC, overnight. The yellowish precipitate was filtered using a Buchner funnel and yielded Fraction A (12 g). Next, dichloromethane was added (three times) to acidic solution in a Buchner flask and partitioned in a separating funnel. Combined dichloromethane parts, were evaporated and concentrated under reduced pressure to yield Fraction B (16 g). Aqueous residue (upper phase in separating funnel) become alkaline by addition of ammonia to reach pH of 8 and left for 30 min. It was partitioned and extracted using dichloromethane (3 × 300 mL) in a separating funnel. Organic layer was evaporated and the resultant was called fraction C (13 g). Aqueous upper phase was adjusted to pH 3 by HCl (0.2 N). 

Mayer's reagent was made by dissolving HgCl_2_ (27.2 g) in a solution of potassium iodide (100 g) in water (2 L). It was added to acidic solution until a yellowish precipitate was produced. Remaining alkaloids were precipitated from acidic solution as a complex of tetraiodomercurate alkaloid in this stage. Tetraiodomercurate alkaloid complex was filtered and washed with small amounts of cold H_2_O and then suspended in a mixture of methanol: water (1:1) and IRA-400 Amberlite resin (previously washed with HCl). Excess Amberlite resin was added until the precipitate was dissolved. In this stage, tetraiodomercurate anion was exchanged with Cl^-^, and it bound to the Amberlite resin. Finally, the mixture was filtered and a clear solution containing alkaloid chloride salts was evaporated under reduced pressure. Concentrated extract was then eluted through a small silica gel column to remove remaining mercuric residues and yield Fraction D (4 g).

Briefly, fraction A, precipitated in acidic aqueous solution, contained quaternary neutral alkaloids predominant in Berberis genus. Fraction B, extracted by dichloromethane from acidic solution contained weak basic alkaloids which are not ionized in dilute HCl. Fraction C, contained free secondary or tertiary basic alkaloids liberated from their chloride salts by alkalinization by ammonia and finally, Fraction D contained remaining alkaloids in alkaloid extract residue (Suau et al., 1998 ▶). 

**Experimental animals**

Male Swiss mice weighing 20-25 g were obtained from animal house of School of Pharmacy, Isfahan University of Medical Sciences (Isfahan, Iran) and housed under standard conditions with 12 hr-12 hr light-dark cycles. Four or five mice were housed in each cage and they had free access to water and food. The animals were transferred to laboratory 2 days before starting the experiments to acclimatize to the test environment. Animal procedures were performed according to guidelines for the care of experimental animals of Isfahan University of Medical Sciences.

**Acetic acid-induced abdominal writhing response**

This method was performed as previously described (Koster et al., 1995 ▶; Hajhashemi et al., 2012 ▶). Briefly, groups of mice (n=6) received different doses of fraction A (25 and 50 mg/kg), B (50 and 100 mg/kg), C (5 mg/kg), D (50, 100 and 200 mg/kg) or total extract (50, 100 and 200 mg/kg) of *B. integerrima *intraperitoneally (i.p.). Animals in control group received 1% solution tween 80 (10 ml/kg, i.p.) while animals in reference group received indomethacin (10 mg/kg, i.p.). After 30 min, 10 ml/kg of 1% acetic acid was injected i.p. and the number of writhes was counted during a 10-min period started 10 min after acetic acid injection.

**Formalin test**

Formalin test was carried out as described by Choi et al. (2001) ▶. Mice were intraperitoneally pretreated with different doses of fractions or total extract of *B. integerrima*, morphine (10 mg/kg) as the standard analgesic drug and 1% Tween 80 as vehicle (10 ml/kg). Thirty minutes later, 20 µl of 2.5% formalin was injected into the subplantar space of the right hind paw of each mouse. The duration of licking the paw was determined during the 1^st^ phase (0–5 min after formalin administration) and the 2^nd^ phase (20–40 min after formalin administration). 

**Hot-plate test**

Mice (n=6/group) were pretreated with vehicle (10 ml/kg i.p.), morphine (10 mg/kg i.p.), different doses of fractions and total extract of *B. integerrima*. Thirty minutes later they were placed on a hot plate maintained at 55 °C. Latency periods were measured 0, 30, 60, 90 and 120 min after the first thermal stimulus. In order to avoid injury to the animals, maximal time of standing on the plate was limited to 30 sec (cut-off time) (Hajhashemi and Khanjani, 2014 ▶). To calculate percent of maximal possible antinociceptive effect (MPE %) we used the following formula: 

MPE%= [test latency (sec)-control latency (sec)]/ [cut-off time (sec) - control latency (sec)] ×100

Test latency: The time period (sec) between exposure to heat stimulus and licking of the hind or front paws after drug treatment.

Control latency: The reaction time before drug treatment.

Cut-off time: Reaction time considered as maximal response. 

**Statistical analysis**

Data were analyzed by SPSS (version 21) using one way analysis of variance (ANOVA) followed by Tukey’s. The results are presented as mean±S.E.M and p-values less than 0.05 were considered significant.

## Results

**Acetic acid-induced writhing test**

**Figure 1:**
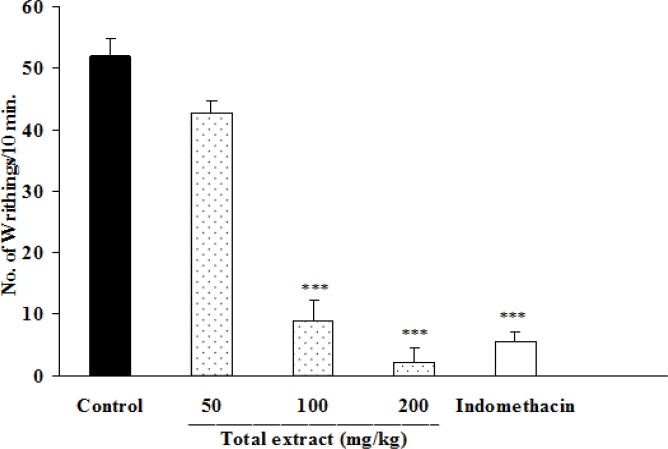
Effect of i.p. injection of total extract of *B. integerrima* on acetic acid-induced writhing test in mice. Vehicle, total extract and indomethacin (10 mg/kg) were administered 30 min prior to acetic acid (1%) injection. The number of abdominal contractions (writhings) was counted for each mouse for a period of 10 min starting from 10 min after acetic acid administration. The values represent the mean±SEM of six mice in each group. *** shows p<0.001 compared to control group

**Figure 2:**
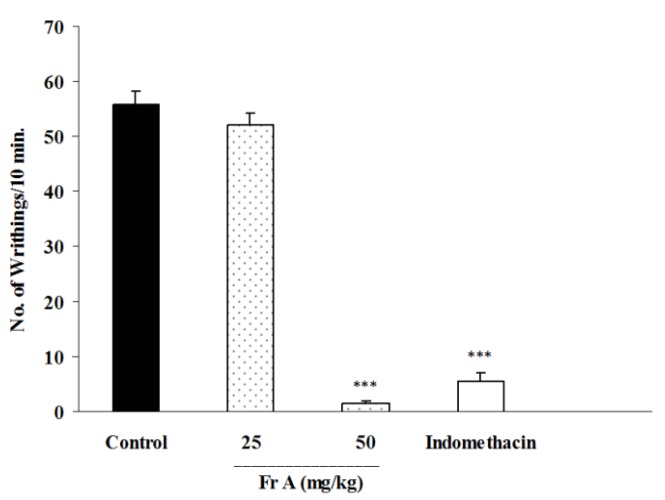
Effect of i.p. injection of fraction A of *B. integerrima* root on acetic acid-induced writhing test in mice. Vehicle, faction A (25 and 50 mg/kg) and indomethacin (10 mg/kg) were administered 30 min prior to acetic acid (1%) injection. The number of abdominal contractions (writhings) was counted for each mouse for a period of 10 min starting from 10 min after acetic acid administration. The values represent the mean±SEM of six mice in each group. *** shows p<0.001 compared to control group

**Figure. 3:**
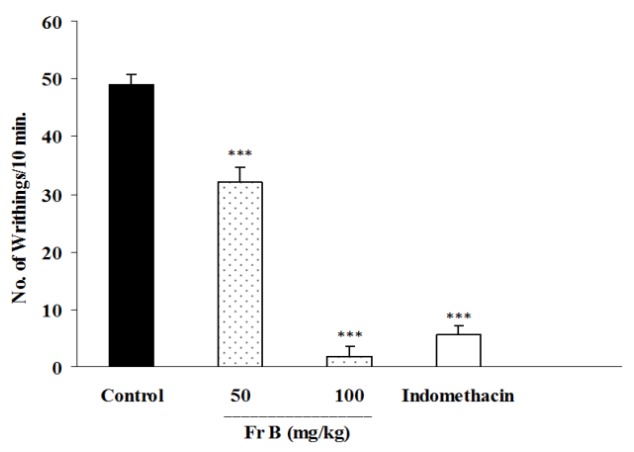
Effect of i.p. injection of fraction B of *B. integerrima* root on acetic acid-induced writhing test in mice. Vehicle, faction B (50 and 100 mg/kg) and indomethacin (10 mg/kg) were administered 30 min prior to acetic acid (1%) injection. The number of abdominal contractions (writhings) was counted for each mouse for a period of 10 min starting from 10 min after acetic acid administration. The values represent the mean±SEM of six mice in each group. *** shows p<0.001 compared to control group

In this test, total alkaloid extract of *B. integerrima *at doses of 100 and 200 mg/kg inhibited abdominal twitches by 83 and 95.7%, respectively (p<0.001 for both cases) (Figure 1). The animals which were pretreated with 50 mg/kg of fraction A (Figure 2), or 50 and 100 mg/kg of fraction B (Figure 3) showed significant reductions (p<0.001 in comparison with control groups) in the numbers of abdominal twitches (writhing) with 97.1, 34.9 and 96.3% of inhibition, respectively. 

**Figure. 4:**
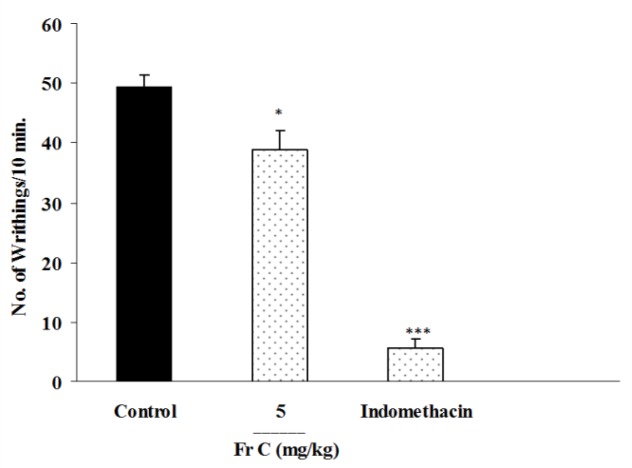
Effect of i.p. injection of fraction C of *B. integerrima* root on acetic acid-induced writhing test in mice. Vehicle, faction C (5 mg/kg) and indomethacin (10 mg/kg) were administered 30 min prior to acetic acid (1%) injection. The number of abdominal contractions (writhings) was counted for each mouse for a period of 10 min starting from 10 min after acetic acid administration. The values represent the mean±SEM of six mice in each group.* p<0.05 and *** show p<0.001 compared to control group

Fraction C at a dose of 5mg/kg showed a slight but significant inhibition of abdominal twitches (Figure 4); however, 10 mg/kg of fraction C caused severe toxicity and even mortality. Fraction D at the doses of 100 and 200 mg/kg reduced abdominal twitches by 65 and 66.1%, respectively while indomethacin as the standard analgesic drug inhibited twitches by 90.13%. 

**Figure. 5.**
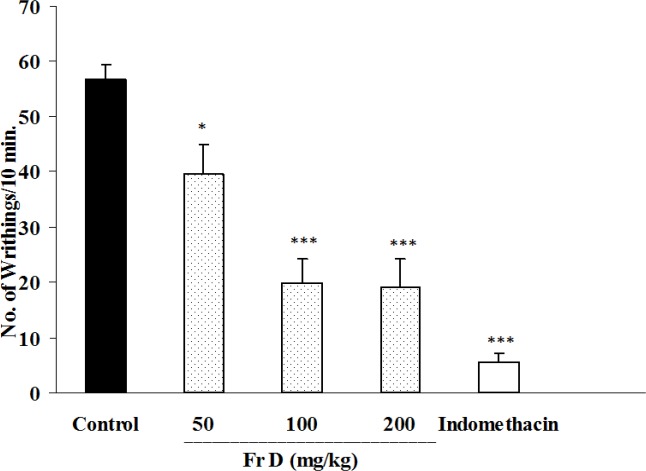
Effect of i.p. injection of fraction D of *B. integerrima* root on acetic acid-induced writhing test in mice. Vehicle, faction D (50, 100 and 200 mg/kg) and indomethacin (10 mg/kg) were administered 30 min prior to acetic acid (1%) injection. The number of abdominal contractions (writhings) was counted for each mouse for a period of 10 min starting from 10 min after acetic acid administration. The values represent the mean±SEM of six mice in each group. * p<0.05 and *** show p<0.001 compared to control group

**Formalin test**

**Figure 6.**
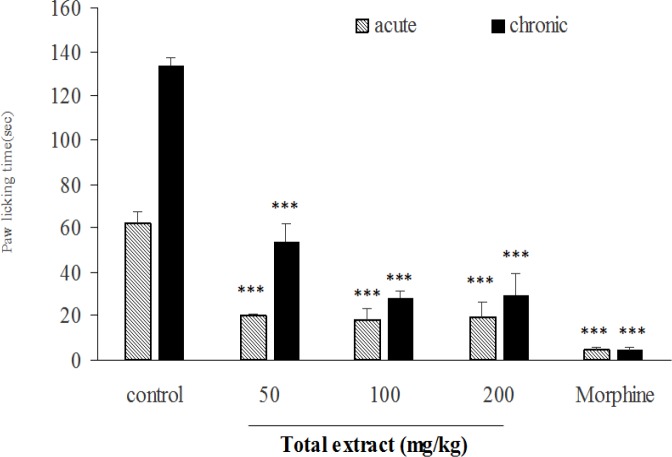
The antinociceptive activity of total extract of *B. integerrima* in acute and chronic phases of formalin test. Different doses of total extract (50, 100, 200 mg/kg) and vehicle (10 ml/kg) were intraperitoneally administered 30 min prior to subplantar injection of formalin and time spent for licking was measured during acute (0-5 min after formalin injection) and chronic phase (20-40 min after formalin injection). Morphine (10 mg/kg, i.p.) was used as a standard drug. Data are expressed as mean±SEM of six animals per each group. *** shows p<0.001 compared to control group

**Figure. 7:**
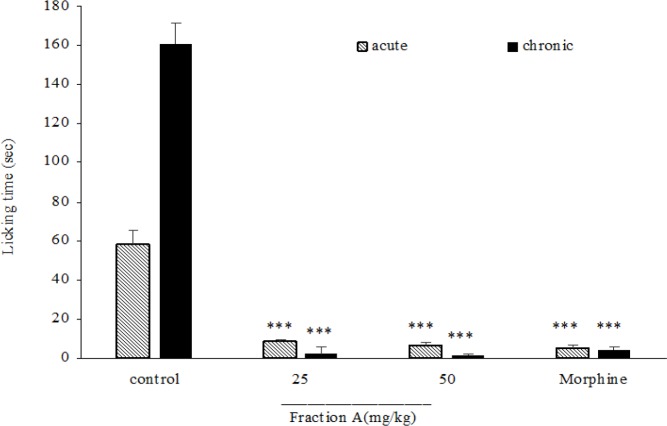
The antinociceptive activity of fraction A of *B. integerrima* in acute and chronic phases of formalin test. Two doses of total extract (25 and 50 mg/kg) and vehicle (10 ml/kg) were intraperitoneally administered 30 min prior to subplantar injection of formalin and time spent for licking was measured during acute (0-5 min after formalin injection) and chronic phase (20-40 min after formalin injection). Morphine (10 mg/kg, i.p.) was used as a standard drug. Data are presented as mean±SEM of six animals per each group. *** shows p<0.001 compared to control group

**Figure 8:**
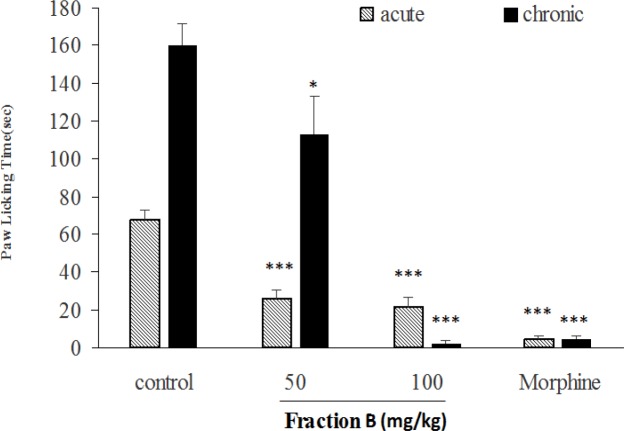
The antinociceptive activity of fraction B of *B. integerrima* in acute and chronic phases of formalin test. Two doses of fraction B (50 and 100 mg/kg) and vehicle (10 ml/kg) were intraperitoneally administered 30 min prior to subplantar injection of formalin and time spent for licking was measured during acute (0-5 min after formalin injection) and chronic phase (20-40 min after formalin injection). Morphine (10 mg/kg, i.p.) was used as a standard drug. Data are presented as mean±SEM of six animals per each group. * p<0.05 and *** show p<0.001 compared to control group

**Figure. 9:**
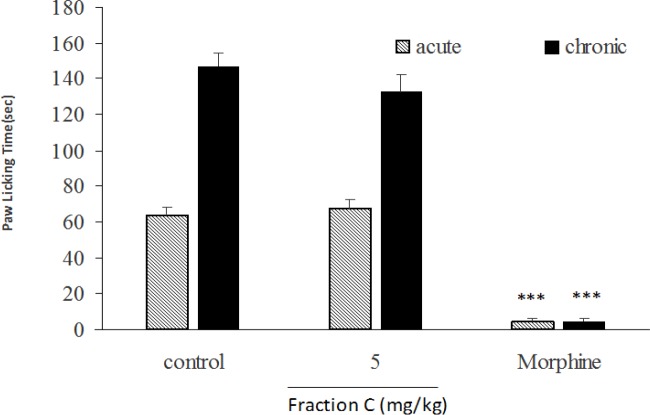
Effect of fraction C of *B. integerrima* on acute and chronic phases of formalin test. Fraction C (5 mg/kg), morphine (10 mg/kg) and vehicle (10 ml/kg) were intraperitoneally administered 30 min prior to subplantar injection of formalin and time spent for licking was measured during acute (0-5 min after formalin injection) and chronic phase (20-40 min after formalin injection). Data are presented as mean±SEM of six animals per each group. *** shows p<0.001 compared to control group

**Figure 10:**
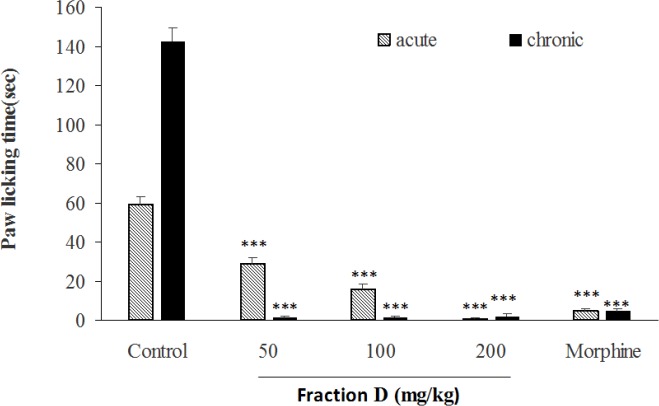
The antinociceptive activity of fraction D of *B. integerrima* in acute and chronic phases of formalin test. Different doses of total extract (50, 100 and 200 mg/kg), morphine (10 mg/kg) and vehicle (10 ml/kg) were intraperitoneally administered 30 min prior to subplantar injection of formalin and time spent for licking was measured during acute (0-5 min after formalin injection) and chronic phase (20-40 min after formalin injection). Data are expressed as mean±SEM of six animals per each group. *** shows p<0.001 compared to control group

In acute and chronic phases of formalin test, the total extract at doses of 50, 100 and 200 mg/kg (Figure 6), fraction A at doses of 25 and 50 mg/kg (Figure 7), fraction B at dose of 100 mg/kg (Figure 8), fraction D at doses of 50, 100 and 200 mg/kg (Figure 10) significantly (p<0.001) inhibited formalin-induced pain. Fraction C at a dose of 5 mg/kg did not show anti-nociception (Figure 9) and larger doses were not tested because of high toxicity. Morphine (10 mg/kg) as a standard analgesic drug significantly (p<0.001) reduced pain in both phases. 

**Hot-plate test**

**Figure 11:**
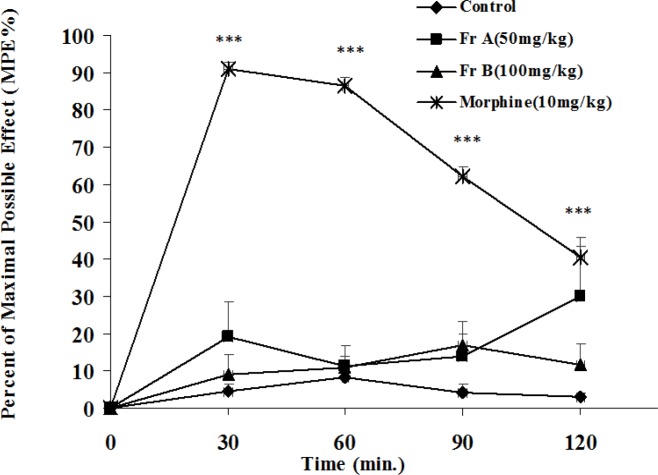
The antinociceptive activity of fractions A and B of *B. integerrima* in hot-plate test. Vehicle and fractions were administered 30 min prior to placement of the animal on hot-plate and reaction time of mice was measured at 30-min intervals until 2 hr and MPE% (percent of maximal possible antinociceptive effect) was calculated for each time and compared. Morphine (10 mg/kg, i.p.) was used as reference drug. Data are expressed as mean±SEM of six animals in each group. *** shows p<0.001 compared to control group

**Figure 12:**
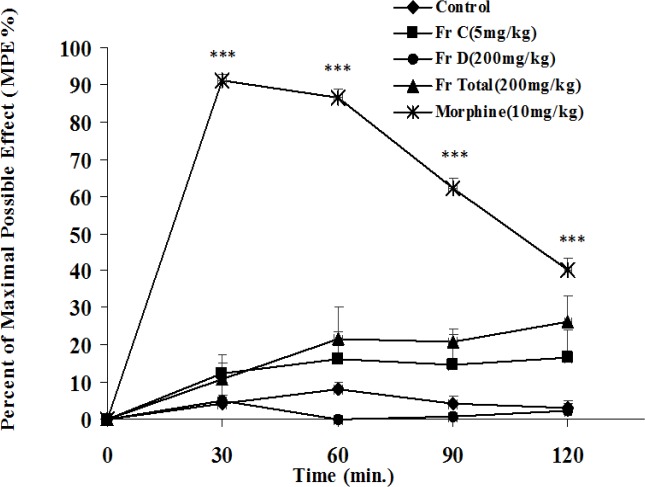
The antinociceptive activity of fractions C and D and total extract of *B. integerrima* in hot-plate test. Vehicle, fractions and total extract were administered 30 min prior to placement of the animal on hot-plate and reaction time of mice was measured at 30-min intervals until 2 hr and MPE% (percent of maximal possible antinociceptive effect) was calculated for each time and compared. Morphine (10 mg/kg, i.p.) was used as reference drug. Data are presented as mean±SEM of six animals in each group. *** shows p<0.001 compared to control group

In hot-plate test, total extract and fractions of *B. integerrima *had no effect on the pain while morphine as the standard drug could significantly (p<0.001) reduce pain (Figures 11 and 12).

## Discussion

In this investigation, total extract and different alkaloid fractions of *B. integerrima *showed analgesic activity in acetic acid-induced writhing and formalin tests but not in hot-plate test.

Following injection of acetic acid into the peritoneal cavity, several mediators including prostaglandins (PGs), bradykinin, histamine, serotonin and substance P are liberated which stimulate primary afferent nociceptors and contribute to the induction of abdominal twitches (Bentley et al., 1983 ▶; Ikeda et al., 2001 ▶; Trongsakul., 2003 ▶). Several classes of drugs, e.g. anticholinergics, calcium channel blockers, non-steroidal anti-inflammatory drugs, opioid analgesics, corticosteroids, some antipsychotic and antidepressant drugs suppress acetic acid nociceptive response and this test is mainly used as a screening method (Vogel and Vogel, 2002 ▶).

The formalin-induced paw licking test is a more valid and reliable model for analgesic activity (Hunskaar and Hole, 1987 ▶). This test has two separate phases. The first phase measures direct chemical stimulation of nociceptors (neurogenic phase), whereas the second phase seems to occur due to an inflammatory response associated with pain that can be inhibited by anti-inflammatory drugs. The acute phase is believed to reflect the activity of C-fiber afferent nociceptors, whereas chronic phase involves histamine, serotonin, PGs, NO and bradykinin (Tjolsen et al., 1992 ▶). Thus, both centrally and peripherally mediated effects can be measured. Total extract of *B. integerrima *and fractions A, B and D at different doses, significantly (p<0.001) reduced licking behavior in this test and these results confirmed the results obtained in acetic acid test. 

Hot-plate test is based on measuring the latency of animals’ reaction to thermal stimuli (Arslan and Bektas, 2010 ▶) and is suitable for measuring the effects of centrally acting analgesic drugs (e.g. opioids) and it is not sensitive to the analgesic effects of nonsteroidal anti-inflammatory agents (Vogel and Vogel, 2002 ▶). Total extract and its fractions at maximal doses which showed effectiveness in acetic acid and formalin tests, could not increase reaction latency in hot-plate test while morphine, as the reference drug significantly demonstrated antinociception and it means that *B. integerrima* root extract and its fractions lack constituents with centrally mediated antinociceptive effect.

The lack of effectiveness of *B. integerrima* in hot-plate test and its considerable analgesic activity in the second phase of formalin test, show that it can suppress pains with inflammatory origins. 

Anti-inflammatory activity has been previously reported for *B. aristata* (Akhter et al., 1977 ▶), *B. vulgaris* (Ivanovska et al., 1996 ▶) and *B. crataegina* (Yesilada and Kupeli, 2002 ▶). Kiasalari et al. (2011) ▶ studied the anti-inflammatry effects of *B. vulgaris *extract on different animal models of acute and chronic inflammation and reported considerable anti-inflammatory effect for the plant extract. Also Minaiyan and colleagues (2011) ▶ reported anti-inflammatory activity for* B. vulgaris* fruit extract in an inflammatory model of ulcerative colitis in rats.

 Also Yeilada and Küpeli (2002) ▶ reported analgesic activity for *B. crataegina* DC. root (Yesilada and Kupeli, 2002 ▶). This study, for the first time, reports the antinociceptive effect of *B.* *integerrima* root extract and its alkaloid fractions. As mentioned above, in acetic acid-induced writhing and also in the second phase of formalin test, histamine is involved as a of pain and inflammation mediator. Shamsa et al. (1999) ▶ reported that aqueous extract of *B. vulgaris* has antihistaminic and anticholinergic properties. One of the various pharmacological actions of histamine is releasing IL-6 from T-cells. Histamine also dilates blood vessels via releasing nitric oxide and therefore, causes increased capillary permeability and leakage of fluid into the sites of inflammation (Shamsa et al., 1999 ▶). In this study, we did check the antihistaminic effect of *B. integerrima* and its alkaloid fractions but if it is true for this species, it might have affected the results obtained in the present study. Another important finding of this study was that fraction C was severely toxic at low doses (e.g. 10 mg/kg) and at the same time, it had no analgesic activity; so, it is suggested that total alkaloid fraction which also contains the components of fraction C seems to be unsuitable for human usage while other fractions have good potential to be regarded as herbal agents with analgesic properties to be used agents especially against inflammatory pain. 

In conclusion, *B. integerrima* and its alkaloid fractions have anti-nociceptive activity and it seems that this effect is peripherally mediated because they are effective in acetic acid and formalin test but not in hot-plate test. Further studies are needed to clarify the exact mechanism of action and also to determine the compounds responsible for these effects. 
